# Risk Factors Associated with COVID-19 Mortality in the State of Durango, Mexico

**DOI:** 10.7150/ijms.82591

**Published:** 2023-05-27

**Authors:** Sebastián Eduardo González Villarreal, Sergio Manuel Salas Pacheco, Fernando Vázquez Alaniz, María Irene Betancourt Conde, José Manuel Salas Pacheco, Karla Cecilia Castillo Vazquez, Alma Cristina Salas Leal, Omar Alejandro Tremillo Maldonado, Juan Antonio Rojas Contreras, Erik Iván Hernández Cosain, Mónica García Montelongo

**Affiliations:** 1 Laboratorio Estatal de Salud Pública de Durango, Secretaría de Salud, Servicios de Salud de Durango, Zip Code 34206, Durango, México.; 2 Unidad de Investigación, Hospital General 450. Servicios de Salud, Zip Code 34206, Durango, México.; 3 Instituto de Investigación Científica, Universidad Juárez del Estado de Durango, Zip Code 34000, Durango, México.; 4 Departamento de investigación, Facultad de Odontología, Universidad Juárez del Estado de Durango, Zip Code 34070, Durango, México.; 5 Dpto. de Ings. Química y Bioquímica, UPIDET, Instituto Tecnológico de Durango, Zip Code 34080, Durango, México.; 6 Facultad de Medicina y Nutrición, Universidad Juárez del Estado de Durango, Zip Code 34000, Durango, México.

**Keywords:** COVID-19, Durango, Comorbidities

## Abstract

The coronavirus disease 2019 (COVID-19) has caused over six million deaths worldwide since its emergence in Wuhan China, factors associated with COVID-19 mortality, such as comorbidities, age, and observed symptomatology still remain a major subject of study. In the present work, a total of 16,345 SARS-CoV-2 positive cases from Durango Mexico diagnosed from May 2020 to December 2021 were analyzed to establish an association of COVID-19 mortality with clinical and demographic variables in a case-control study. Selected variables include patient age, smoking status, sex, presence of comorbidities such as hypertension, diabetes and obesity, as well as patient symptomatology such as fever, dyspnea, abdominal pain and diarrhea. Results indicate that among analyzed data, the median age was 43 years; 54% were female, with a mortality rate of 5.66%. Multivariate regression analysis indicated that the comorbidities associated with the highest risk factor were advanced age (>60) with an odds ratio of 4.127 (IC 95%, 3.37-5.05), hypertension with 1.961 (IC 95%, 1.57-2.45), diabetes with 1.753 (IC 95%, 1.39-2.20) and obesity with 1.413 (IC 95%, 1.11-1.78) respectively. On the other hand, the symptom associated with the highest risk factor was dyspnea with an odds ratio of 18.369 (IC 95%, 14.42-23.39). Our data suggests an association between hypertension and old age with COVID-19 mortality. Other findings include the prevalence of dyspnea, polypnea and cyanosis as a major predictor for COVID-19 mortality, as well as lower mortality risks among health workers.

## Introduction

After its first report in late 2019 in Wuhan China, the 2019 novel coronavirus (2019-nCoV) quickly spread throughout the world causing the 2020 pandemic. COVID-19, which is caused by the 2019-nCoV, current namely, severe acute respiratory syndrome coronavirus 2 (SARS-CoV-2) 5, exhibits diverse levels of severity and a varied array of symptoms that include: cough, dyspnea, diarrhea, fever or chills, fatigue, muscle aches, headache, shortness of breath, nausea, sore throat and new loss of taste or smell 11. The COVID-19 pandemic has caused over 6,543,268 deaths as of September 2022 15. In Mexico, the COVID-19 mortality rate is the second highest in Latin America after Peru, and among the top 10 worldwide 13 Even though mortality by COVID-19 can be influenced by multiple variables 10, comorbidities associated with obesity, such as diabetes and hypertension, have been reported to play a significant role in the outcome of COVID-19 infection, Lippi *et al*., 9 for example, determined that hypertension increased 2.5 fold the risk of increased severity and mortality by COVID-19, diabetes on its part, was twice as prevalent in COVID-19 victims from China and Italy 6; 16. Patient age and sex also appear to contribute to COVID-19 mortality, with ages ≥50 having an increased 15.4-fold risk of mortality compared to ages <50 3, lastly, male patients exhibit twice the likelihood of dying compared to female patients 8. Mexico faces a major public health concern regarding obesity, where over 71% of the population is overweight or obese 7. As expected, many ailments associated with obesity are also present in the Mexican population; diabetes deaths in 2020 corresponded to 14% of the total deaths (INEGI). Hypertension is, as of 2020, present in approximately 25% of the adult population, with 50,000 deaths associated to it each year (INEGI). The objective of this study is to determine if age, sex, occupation, symptomatology, and comorbidities influence the risk of mortality in patients diagnosed with COVID-19 in the state of Durango, Mexico.

## Materials and Methods

Single-center and cross-sectional study of 16,345 people residing in Durango, Mexico with positive result for SARS-CoV-2 confirmed by RT-PCR performed at the State Public Health Laboratory of Durango from December 2020 to December 2021, mortality represented 5.7% of the cases. Data collection about demographic characteristics as: occupation, age, sex, coexisting chronic illness (e.g. hypertension, diabetes, renal impairment, asthma and/or chronic obstructive pulmonary disease, obesity, cardiovascular disease, hepatic impairment, cancer, and disorders associated with immune dysfunction as HIV, other immunosuppressive conditions) and symptomatology was obtained from the National Epidemiological Surveillance System of the Mexican Ministry of Health and performed with Microsoft Office Power Desktop Automate^®^, and data analysis with R Project Studio and IBM ^®^ SPSS^®^ Statistics software V.28.2 related with data was analyzed by multivariate logistic regression to determine the relationship between COVID mortality as dependent variable and socio-demoghraphic and clinical comorbidities as independent variables, the model was created forward including all variables included in Table 4.

## Results

**Figure 1:**
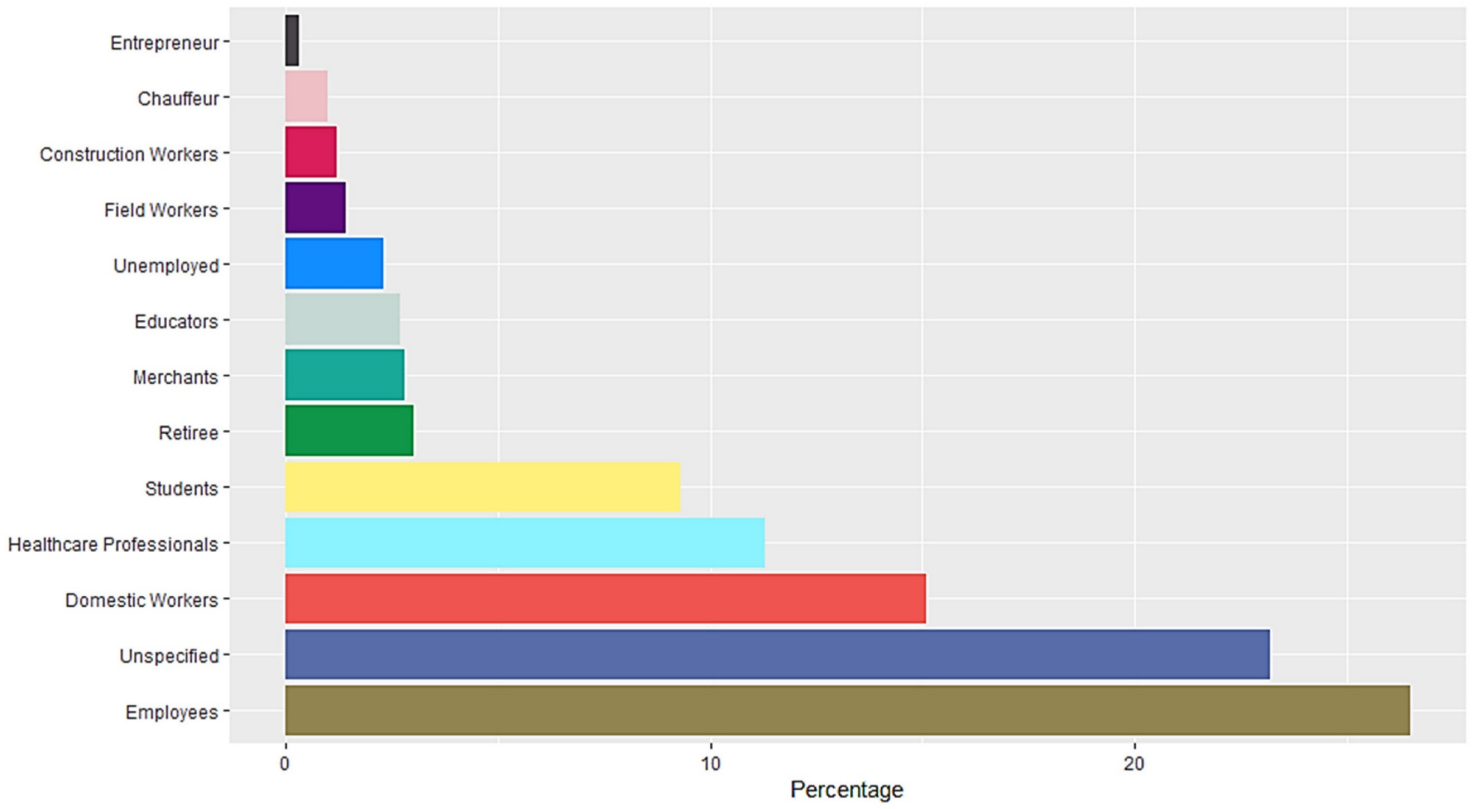
Occupation of RT-SARS-CoV-2 confirmed positive cases

**Figure 2:**
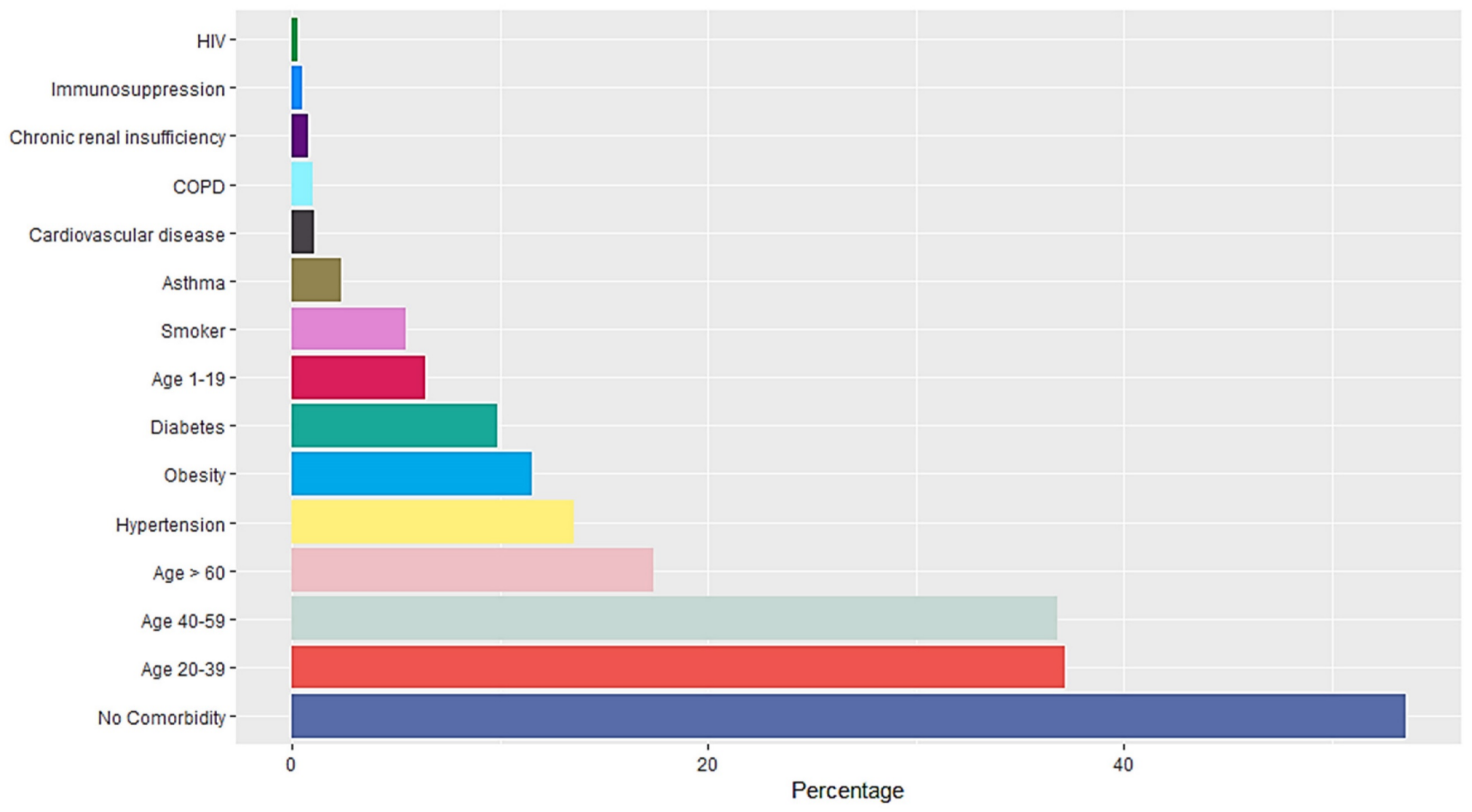
Comorbidities of RT-SARS-CoV-2 confirmed positive cases

**Figure 3:**
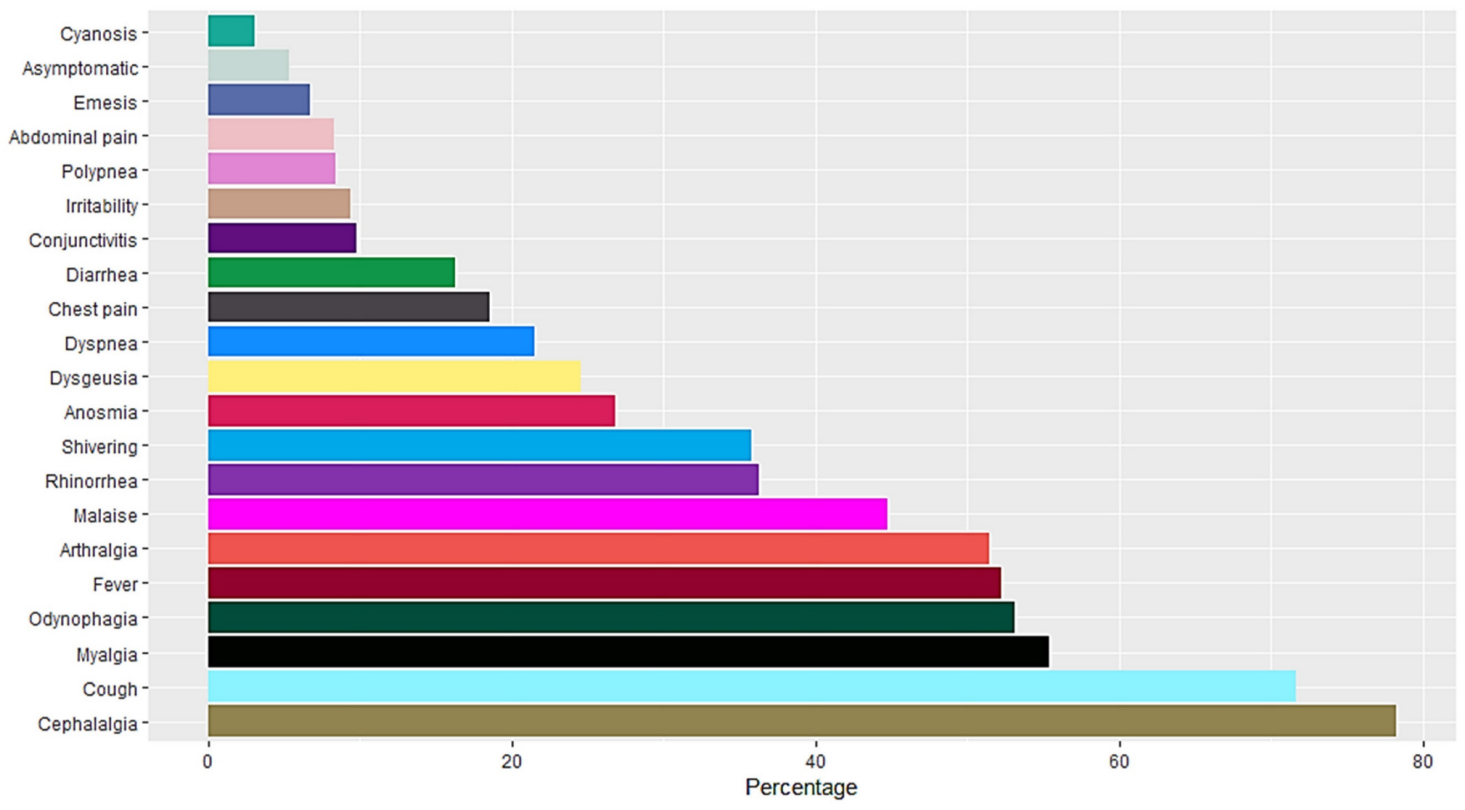
Symptomatology of RT-SARS-CoV-2 confirmed positive cases

A total of 16,345 RT-PCR SARS-CoV-2 confirmed positive cases were analyzed. The mean age population was 45.06 years old with a standard deviation of 15.64, 54% were female and 46% were male. Occupation data included employees, domestic workers, healthcare professionals, students, retirees, merchants, educators, unemployed, field workers, construction workers, chauffeurs, entrepreneurs and unspecified. Students, healthcare professionals, domestic workers and employees represent more than 50% of the confirmed positive cases (Figure 1). Data included Diabetes type II, age, chronic obstructive pulmonary disease (COPD), asthma, immunosuppression, hypertension, HIV, cardiovascular disease, obesity, chronic renal insufficiency, and smoker (Figure 2). Most patients presented no comorbidity, while the most common comorbidities in this cohort were diabetes, hypertension, obesity and age above 60. Symptomatology data included fever, cough, cephalalgia, dyspnea, irritability, diarrhea, chest pain, shivering, odynophagia, myalgia, arthralgia, malaise, rhinorrhea, polypnea, emesis, abdominal pain, conjunctivitis, cyanosis, anosmia, dysgeusia and asymptomatic. Malaise, arthralgia, fever, odynophagia, myalgia, cough and cephalalgia were present in more than 50% of the cases, being cephalalgia the most prevalent symptom, observed in almost 80% of the analyzed cases (Figure 3).

### Occupation and COVID-19 mortality

To assess an association between occupation and mortality among patients, we performed a multivariate logistic regression including 13 self-reported occupations among discharged/deceased cases. Occupations p<0.01 included employees, unspecified, domestic workers, healthcare professionals, students, retiree, educators, unemployed and field workers. The occupations that represented a risk for mortality associated to COVID-19 were domestic work (OR 1.38, CI [1.15-1.66]), unspecified (OR 1.28, CI [1.11-1.49]), unemployment (OR 3.60, CI [2.73-4.75]), retirement (OR 3.90, CI [3.06-4.98]) and field work (OR 4.54, CI [3.30-6.23]). The occupations that acted as a protection factor for mortality associated with COVID-19 were employment (OR 0.217, CI [0.166-0.283]), healthcare professional (OR 0.13, CI [0.08-0.21]), student (OR 0.02, CI [0.00-0.13]) and educator (OR 0.40, CI [0.23-0.71]) as shown in Table 1.

### Comorbidity and COVID-19 mortality

Twelve self-reported comorbidities were included in a multivariate logistic regression model, age was divided in four categories (1-19, 20-39, 40-59 and >60 years of age). The only comorbidities not related to mortality were age 1-19, 20-39, smoking, HIV and immunosuppression. The comorbidities that represented a risk for mortality associated to COVID-19 were age 20-39 (OR 26.9, CI [3.76-192.79]), age >60 (OR 116.10, CI [16.23-830.21]), hypertension (OR 1.357, CI [1.11-1.65]), obesity (OR 1.44, CI [1.18-1.75]), diabetes (OR 1.41, CI [1.17-1.70]), cardiovascular disease (OR 1.52, CI [1.03-2.25]), COPD (OR 1.75, CI [1.20-2.56]), chronic renal insufficiency (OR 2.28, CI [1.41-3.705]) and male gender (OR 1.989, [1.694-2.336]). Variables that acted as protective factors against mortality associated to COVID-19 included having no comorbidity (OR 0.465, CI [0.360-0.602]) and asthma (OR 0.407, CI [ 0.230-0.719]) as shown in Table 2.

### Symptomatology and COVID-19 mortality

Furthermore, 22 symptoms were included in our statistic model. Symptomatology with p<0.05 included cephalalgia, myalgia, odynophagia, arthralgia, malaise, rhinorrhea, anosmia, dyspnea, diarrhea, conjunctivitis, irritability, polypnea, cyanosis and others. Symptomology associated with increased risk of COVID-19 mortality were arthralgia (OR 1.41, CI [1.06-1.88]), malaise (OR 1.57, IC [1.29-1.91]), dyspnea (OR 24.44, IC [19.4-30.8]), polypnea (OR 2.66, IC [2.11-3.36]) and cyanosis (OR 1.70, IC [1.19-2.43]). Symptoms representing a protection factor for mortality related to COVID-19 included cephalalgia (OR 0.581, IC [0.474-0.712]), myalgia (OR 0.709, IC [0.53-0.94]), odynophagia (OR 0.614, IC [0.50-0.75]), rhinorrhea (OR 0.762, IC[0.61-0.94]), anosmia (OR 0.54, IC [0.38-0.76]), diarrhea (OR 0.64, IC [0.50-0.82]), conjunctivitis (OR 0.404, IC [0.28-0.58], irritability (OR 0.722, IC [0.55-0.947]), and others (OR 0.477, IC [0.299-0.762]) as shown in Table 3.

### Comorbidities and symptomatology related to COVID-19 mortality

**Figure 4:**
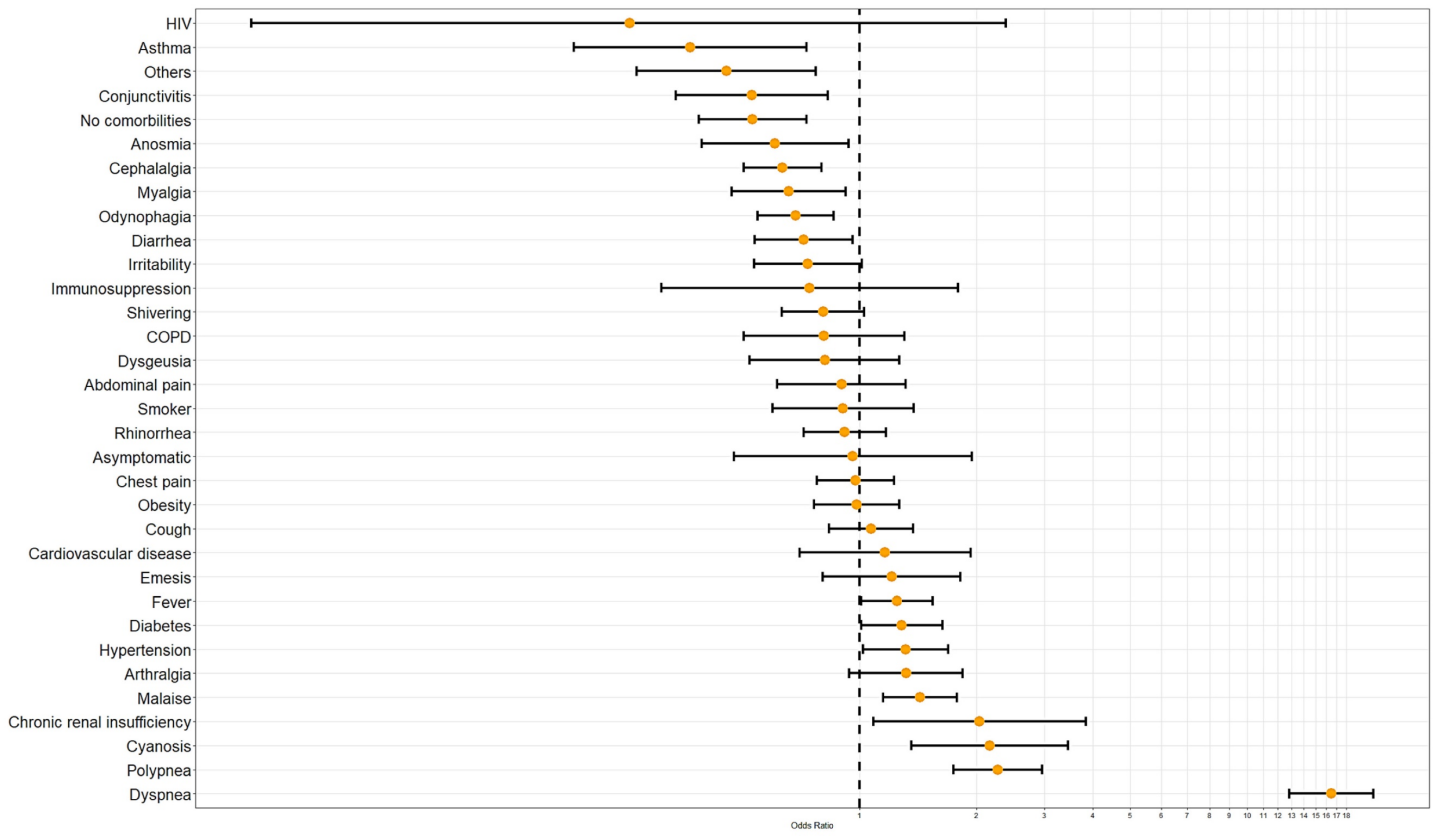
Comorbidities and symptomatology odds ratio of RT-SARS-CoV-2 confirmed positive cases related to mortality risk.

Finally, a last regression model including 12 comorbidities and 22 symptoms was performed. Only 13 maintained statistical significance p<0.01 and seven p<0.05. Dyspnea, polypnea, cyanosis, chronic renal insufficiency, malaise, hypertension, diabetes, and fever are associated with mortality by COVID-19 as a risk factor. In contrast asthma, conjunctivitis, anosmia, cephalalgia, myalgia, odynophagia, diarrhea, having no comorbidities and others presented an association to mortality related to COVID-19 as a protecting factor (Table 4, Figure 4).

## Conclusion and Discussion

Analyzed data suggests that occupations associated with COVID-19 mortality were those that involved high/moderate amounts of physical labor (such as field work and domestic work) and advanced age (retirement). As previously shown, age constitutes an important variable regarding the evolution of COVID-19 infections, as age increases, the risk of mortality increases as well, and 60 years old sets up the highest risk according to our results. Intense physical activities are considered dangerous during COVID-19 infection and advised to be avoided 12, this could also explain why activities with low physical activity (employees, students, educators) resulted protective against COVID-19 mortality. Even though, one could assume that health workers would be at a greater risk of experiencing mortality, our data suggests that this occupation possessed a protection factor against COVID-19, we hypothesize that this is due to lower comorbidity rates (approximately 20% below the comorbidity median), swiftness of diagnostic, earlier access to treatment and awareness health workers had during the pandemic 2. Gender also plays an important role as a risk factor, of all mortality cases 61% were males, this supports the evidence shown in other studies where male patients exhibited two times the risk of covid related mortality 1.

Of the selected comorbidities, hypertension presented the most prevalent association with mortality, this result is in accordance with previous research regarding the role of the blood pressure homeostasis enzyme mACE2 (Membrane bound Angiotensin-converting enzyme 2) which acts as main entry point for SARS-CoV-2 into the cell, and how blood pressure alterations influence the pathologic course of COVID-19 14.

Cytokine storm syndrome, which is associated with severe COVID-19 infections, causes a rapid increase in cytokines that attract an excess of immune cells that infiltrate into lung tissue causing lung injury, subsequent failure and ultimately death of the infected individual 17. Our results indicate a link between symptoms associated to the respiratory system and mortality, dyspnea being the most prevalent symptom associated with COVID-19 mortality, additional symptoms related to alterations of the respiratory system such as cyanosis and polypnea also influence mortality. Other factors determinant in the course of COVID-19 infection appear to be age and sex 4, our data suggests that ages >60 present the highest mortality risk.

We consider that these results contribute to the ever growing data regarding the close relationship between COVID-19 mortality and patient characteristics, as expected, obesity related comorbidities, mainly hypertension, play a major role not only in COVID-19 outcome, but in a plethora of other diseases as well, countries with high obesity rates (such as Mexico) need to always keep this information present in order to develop new public health strategies. It is worth highlighting that results regarding occupation and predictor symptomatology factors were of great interest in this study, as they can provide valuable information for the surveillance and care of patients who perform high risk jobs and/or exhibit symptoms associated with unfavorable COVID-19 outcomes. Durango City initially focused most of its public health strategies to health professionals, however, our results indicate that even though this occupation has the highest COVID-19 positive rates, it also possesses a very low mortality risk.

Data included in our study did not consider the condition of patients and quality of data post-infection. All data was extracted from databases and not corroborated with each patient, these factors the most important limitations of our study.

## Figures and Tables

**Table 1:** Occupation of SARS-CoV2 positive cases

Occupation	(%)	p	Exp (B)	95% C.I. for EXP(B)	
				Lower	Upper
**Employees**	26.5	0.000	0.217	0.166	0.283
**Unspecified**	23.2	0.000	1.287	1.110	1.492
**Domestic Workers**	15.1	0.001	1.38	1.15	1.66
**Healthcare Professionals**	11.3	0.000	0.13	0.08	0.21
**Students**	9.3	0.000	0.02	0.00	0.13
**Retiree**	3	0.000	3.90	3.06	4.98
**Merchants**	2.8	0.889	1.03	0.71	1.49
**Educators**	2.7	0.002	0.40	0.23	0.71
**Unemployed**	2.3	0.000	3.60	2.73	4.75
**Field Workers**	1.4	0.000	4.54	3.30	6.23
**Construction Workers**	1.2	0.210	1.38	0.83	2.28
**Chauffeur**	1	0.062	1.62	0.98	2.69
**Entrepreneur**	0.3	0.997	0.00	0.00	0.00
**Constant**			0.000		

**Table 2:** Comorbidity of SARS-CoV2 positive cases related to mortality risk.

Comorbidity	(% cases)	P	Exp (B)	95% C.I. for EXP(B)	
				Lower	Upper
**No Comorbidity**	53.6	0.000	0.472	0.364	0.611
**Gender (Male)**	46.1	0.000	1.989	1.694	2.336
**Age 20-39**	37.2	0.123	4.804	0.655	35.238
**Age 40-59**	36.8	0.001	27.564	3.853	197.191
**Age > 60**	17.4	0.000	119.019	16.644	851.088
**Hypertension**	13.6	0.001	1.422	1.163	1.739
**Obesity**	11.5	0.000	1.602	1.315	1.953
**Diabetes**	9.9	0.000	1.453	1.204	1.755
**Age 1-19**	8.6	0.143	0.225	0.031	1.653
**Smoker**	5.5	0.651	0.929	0.676	1.278
**Asthma**	2.4	0.007	0.458	0.259	0.810
**Cardiovascular disease**	1.1	0.082	1.411	0.957	2.080
**COPD**	1.0	0.003	1.756	1.204	2.562
**Chronic renal insufficiency**	0.8	0.001	2.256	1.392	3.655
**Immunosuppression**	0.5	0.223	1.513	0.777	2.948
**HIV**	0.3	0.074	0.156	0.020	1.198
**Constant**			0.000		

**Table 3:** Symptomatology of SARS-CoV2 positive cases related to mortality risk

Symptoms	%	P	Exp(b)	95% C.I. for EXP(B)	
				Lower	Upper
**Cephalalgia**	77.6	0.000	0.581	0.474	0.712
**Cough**	71.0	0.480	1.082	0.87	1.346
**Myalgia**	54.0	0.017	0.709	0.535	0.94
**Odynophagia**	52.7	0.000	0.614	0.503	0.75
**Fever**	52.1	0.053	1.203	0.998	1.45
**Arthralgia**	50.0	0.017	1.414	1.063	1.88
**Malaise**	43.7	0.000	1.574	1.294	1.915
**Rhinorrhea**	36.0	0.012	0.762	0.616	0.942
**Shivering**	34.9	0.149	0.855	0.691	1.058
**Anosmia**	26.2	0.001	0.543	0.383	0.768
**Dysgeusia**	23.9	0.083	0.732	0.514	1.041
**Dyspnea**	20.5	0.000	24.444	19.4	30.8
**Chest pain**	17.8	0.672	1.045	0.851	1.283
**Diarrhea**	15.8	0.001	0.644	0.501	0.827
**Conjunctivitis**	9.6	0.000	0.404	0.28	0.582
**Irritability**	9.1	0.018	0.722	0.551	0.947
**Abdominal pain**	8.1	0.405	0.873	0.635	1.201
**Polypnea**	8.1	0.000	2.667	2.116	3.362
**Emesis**	6.5	0.440	1.142	0.815	1.598
**Others**	6.1	0.002	0.477	0.299	0.762
**Asymptomatic**	5.0	0.247	0.666	0.335	1.324
**Cyanosis**	3.0	0.004	1.702	1.19	2.434
**Constant**		0			

**Table 4:** Comorbidities and symptomatology of SARS-CoV2 positive cases related to mortality risk

Comorbidity or Symptoms	p-value ^*^	Exp(B)	95% C.I. for EXP(B)	
			Lower	Upper
**Asymptomatic**	0.911	0.960	0.474	1.947
**Fever**	0.041	1.247	1.009	1.542
**Cough**	0.596	1.070	0.834	1.373
**Cephalalgia**	0.000	0.632	0.502	0.797
**Dyspnea**	0.000	16.475	12.838	21.142
**Irritability**	0.060	0.735	0.534	1.014
**Diarrhea**	0.025	0.718	0.537	0.960
**Chest pain**	0.841	0.977	0.777	1.229
**Shivering**	0.082	0.805	0.630	1.028
**Odynophagia**	0.001	0.683	0.545	0.858
**Myalgia**	0.015	0.656	0.467	0.920
**Arthralgia**	0.108	1.318	0.941	1.847
**Malaise**	0.001	1.430	1.149	1.780
**Rhinorrhea**	0.481	0.916	0.718	1.169
**Polypnea**	0.000	2.273	1.748	2.956
**Emesis**	0.365	1.209	0.802	1.822
**Abdominal pain**	0.579	0.898	0.613	1.315
**Conjunctivitis**	0.005	0.527	0.336	0.827
**Cyanosis**	0.001	2.164	1.359	3.445
**Anosmia**	0.025	0.605	0.391	0.938
**Dysgeusia**	0.360	0.813	0.521	1.267
**Others**	0.004	0.453	0.266	0.771
**Age > 60 years**	0.000	46.765	6.381	342.750
**Age 40-59 years**	0.006	16.131	2.202	118.185
**Age 20-39 years**	0.150	4.407	0.586	33.133
**Non comorbidities**	0.000	0.530	0.385	0.731
**Diabetes**	0.041	1.285	1.010	1.634
**COPD**	0.387	0.810	0.502	1.306
**Asthma**	0.004	0.366	0.183	0.730
**Immunosuppression**	0.508	0.743	0.308	1.793
**Hypertension**	0.035	1.314	1.019	1.694
**HIV**	0.230	0.255	0.027	2.381
**Cardiovascular disease**	0.559	1.163	0.700	1.933
**Obesity**	0.900	0.984	0.764	1.267
**Chronic kidney disease**	0.027	2.039	1.085	3.834
**Smoker history**	0.644	0.906	0.596	1.377
**Constant**	0.000	0.001		

*p-value corresponds to Odds Ratio (OR) and was calculated by multivariate lineal regression.
